# Supplementary material for: LCRMP-1 is required for spermatogenesis and stabilises spermatid F-actin organization via the PI3K-Akt pathway
Source: Commun Biol. 2023 Apr 10;6:389. doi: 10.1038/s42003-023-04778-2 (PMC10086033; doi:10.1038/s42003-023-04778-2)
Supplement: Supplementary file 2 — Supplementary Information [file 42003_2023_4778_MOESM2_ESM.pdf]

Supplementary Information

LCRMP-1 is required for spermatogenesis and stabilises spermatid F-actin organization via the PI3K-Akt pathway

Jung-Hsuan Chang[1], Chia-Hua Chou[1], Jui-Ching Wu[1], Keng-Mao Liao[2], Wei-Jia Luo[1], Wei-Lun Hsu[1], Xuan-Ren Chen[3], Sung-Liang Yu[1], Szu-Hua Pan[2,3,4], Pan-Chyr Yang[5], and Kang-Yi Su[1,2,6,*]

[1]Department of Clinical Laboratory Sciences and Medical Biotechnology, College of Medicine, National Taiwan University, Taipei, Taiwan

[2]Genome and Systems Biology Degree Program, National Taiwan University and Academia Sinica, Taipei, Taiwan

[3]Graduate Institute of Medical Genomics and Proteomics, College of Medicine, National Taiwan University, Taipei, Taiwan

[4]Doctoral Degree Program of Translational Medicine, National Taiwan University, Taipei, Taiwan

[5]Department of Internal Medicine, National Taiwan University, College of Medicine, Taipei, Taiwan

[6]Department of Laboratory Medicine, National Taiwan University Hospital, Taipei, Taiwan

[*]Correspondence author (Email: suky@ntu.edu.tw)

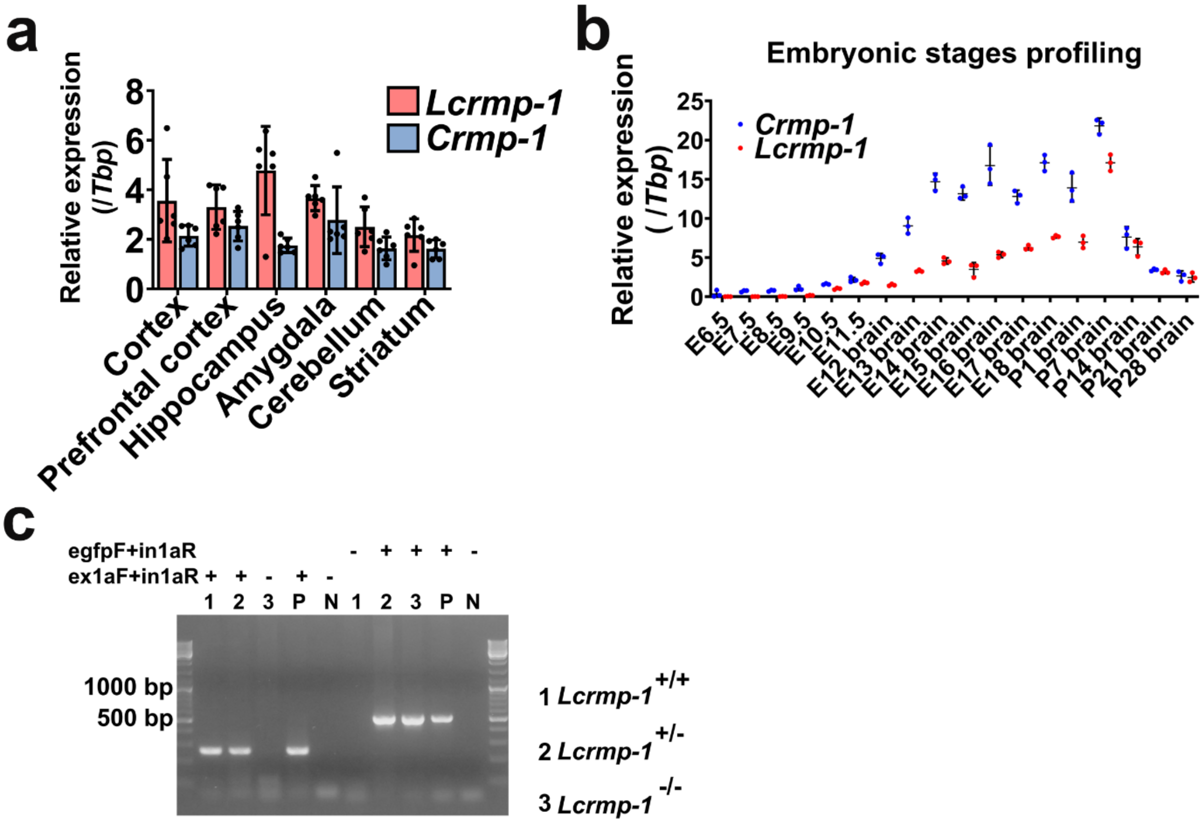

Supplementary Fig. 1 LCRMP-1 expression profiling in the brain and embryonic stages.

a qPCR analysis of LCRMP-1 and CRMP-1 expression in different parts of brain. (n = 6, mean ± SD). **b** Expression profile of LCRMP-1 and CRMP-1 in embryonic and postnatal stages. From E6.5 (days) to E11.5 (days), the RNA of whole embryo was extracted for RT-PCR and qPCR. From E12 (days) to E18 (days), the brain of embryo was isolated for qPCR analysis. (n = 3, mean ± SD). **c** The genotyping strategy of *Lcrmp-1*$^{+/+}$ and *Lcrmp-1*$^{-/-}$ mice. DNA was extracted from mouse tails followed by PCR with corresponding primer pairs. The primer set ex1aF + in1aR was utilized for the detecting *Lcrmp-1*$^{+/+}$ allele while egfpF + in1aR for detecting the *Lcrmp-1*$^{-/-}$ allele. “P” indicates the sample positive control while “N” indicates blank negative control.

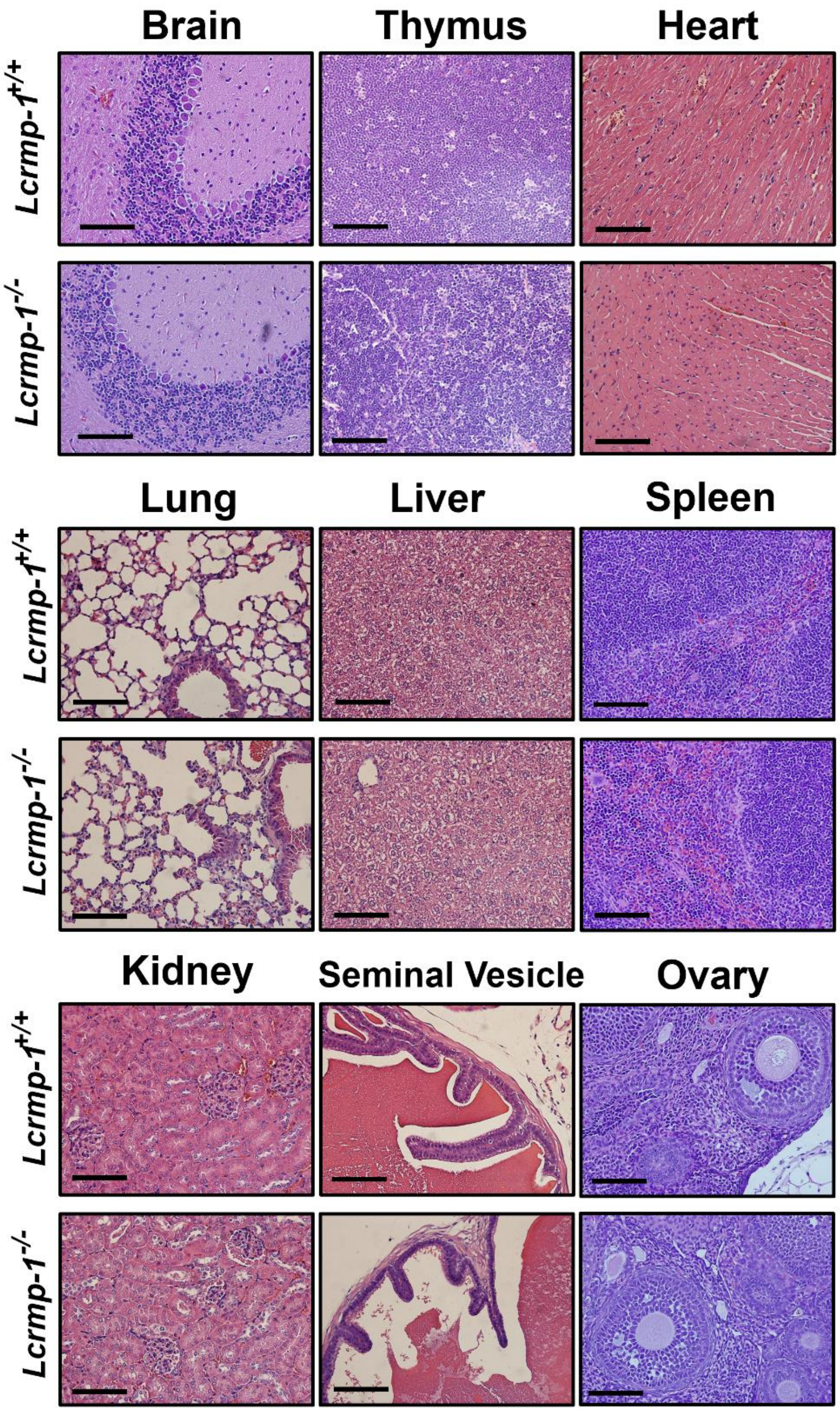

Supplementary Fig. 2 The histology of major organs in *Lcrmp-1*$^{+/+}$ and *Lcrmp-1*$^{-/-}$ mice.

The mice organs were isolated and embedded. Sections were performed by Hematoxylin-eosin staining for histopathological analysis. Scale bars, 100 μm.

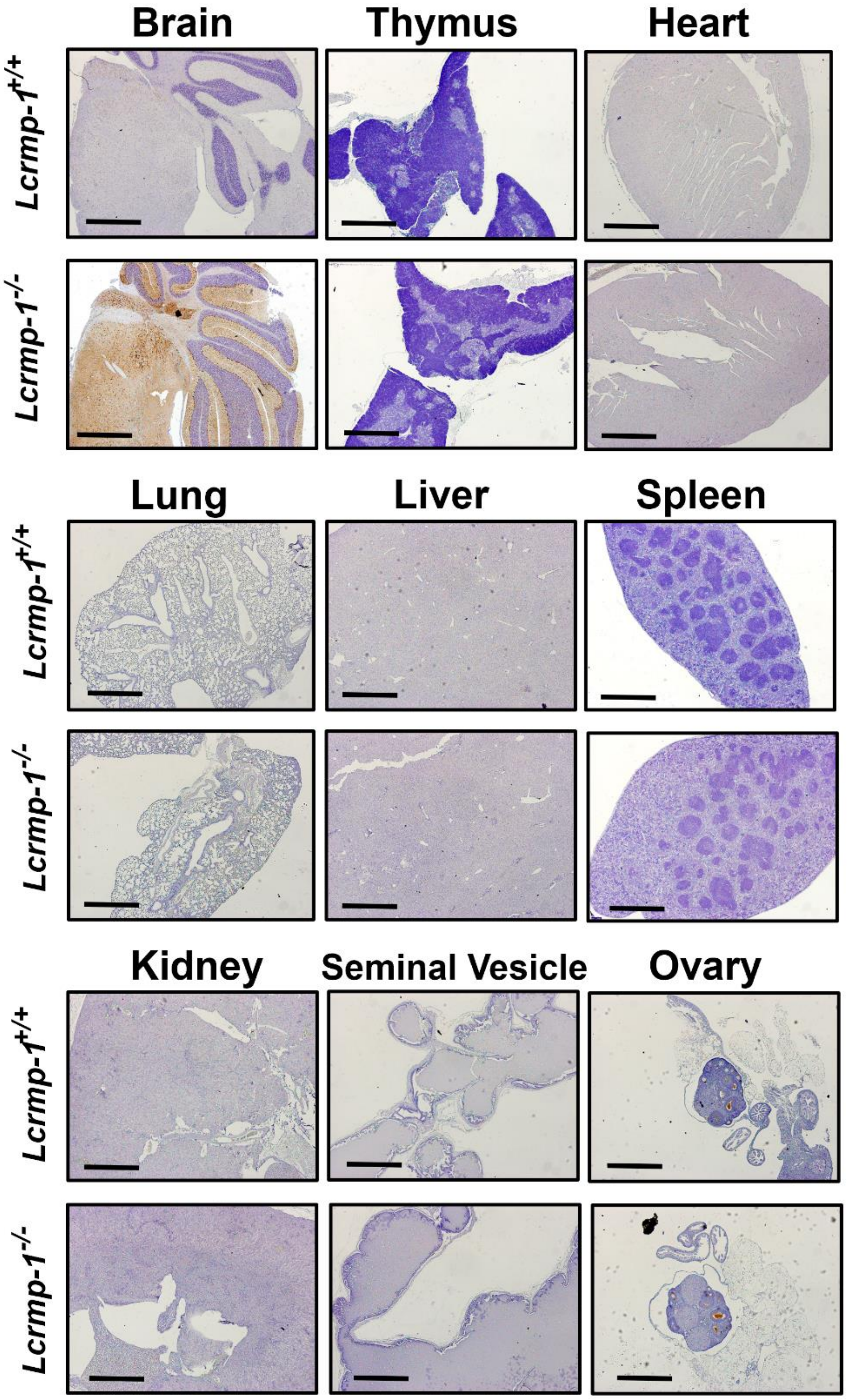

Supplementary Fig. 3 The expression pattern of LCRMP-1 in major organs.

Major organs were isolated and embedded for standard immunohistochemical staining with eGFP antibodies to represent LCRMP-1 expression. Scale bars, 1 mm.

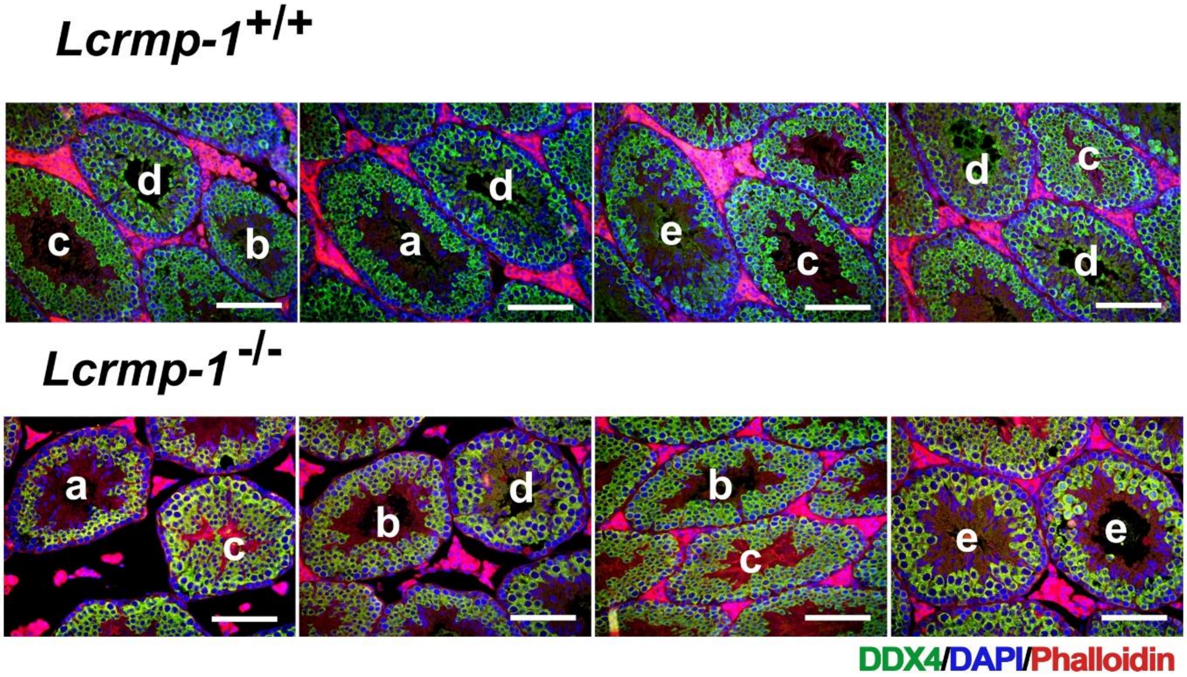

Supplementary Fig. 4 DDX4 expression pattern in testes of *Lcrmp-1*$^{+/+}$ and *Lcrmp-1*$^{-/-}$ mice.

Testes were isolated and embedded for standard immunofluorescence staining with DDX4 antibodies. Stages of spermatogenesis were further identified as a for stages I – III, b for stages IV – VI, c for stages VII – VIII, d for stages IX – X, and e for stages XI – XII. Scale bars, 100 μm.

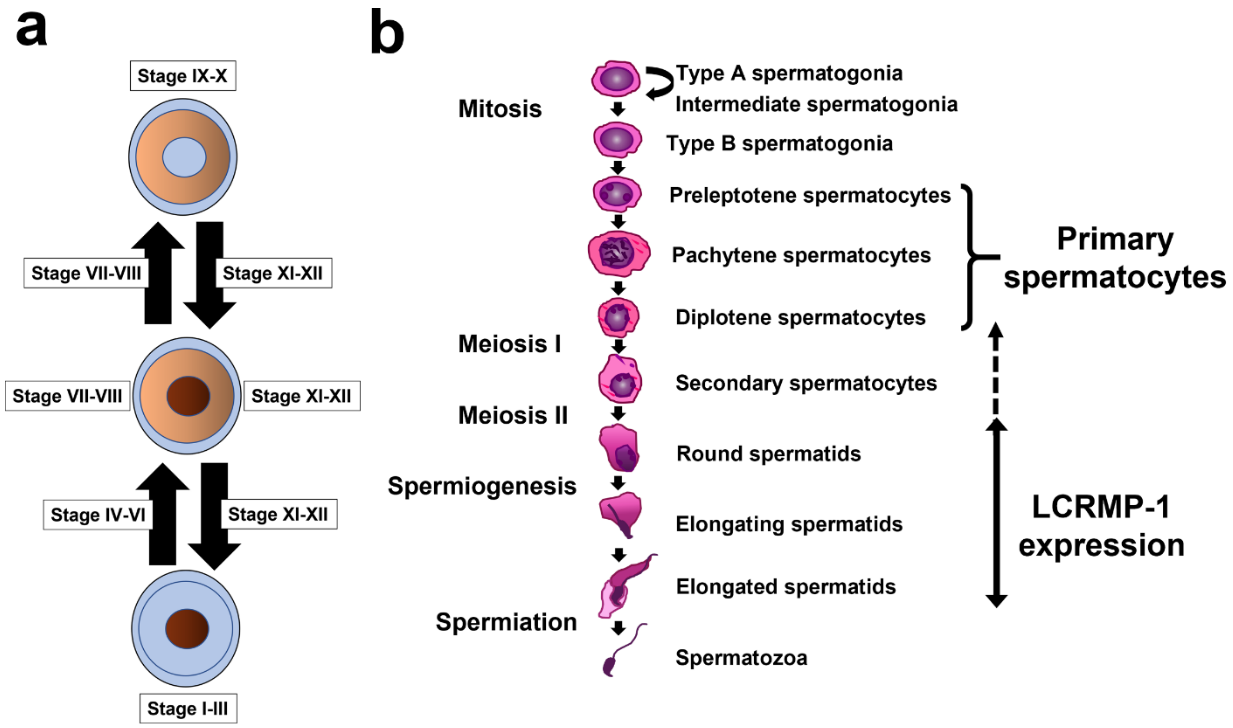

Supplementary Fig. 5 Illustration of cyclic expression of LCRMP-1 in seminiferous tubules.

a The expression of LCRMP-1 (maroon for strong signal, and light brown for weak signal) migrated in the seminiferous tubules from stages I-III, the positive signal concentrate in the center of lumen, to stages VII-VIII and stages IX-XII, the signal "diffused" from the basal to the adluminal compartment of seminiferous tubules. At stages IX-X, the signal appeared in spermatocytes and elongating spermatids above the BTB. **b** LCRMP-1 expression during spermatogenesis. LCRMP-1 was stably expressed in different stages of spermatogenesis (solid arrow) and partially expressed in stages VII-X (dotted arrow).

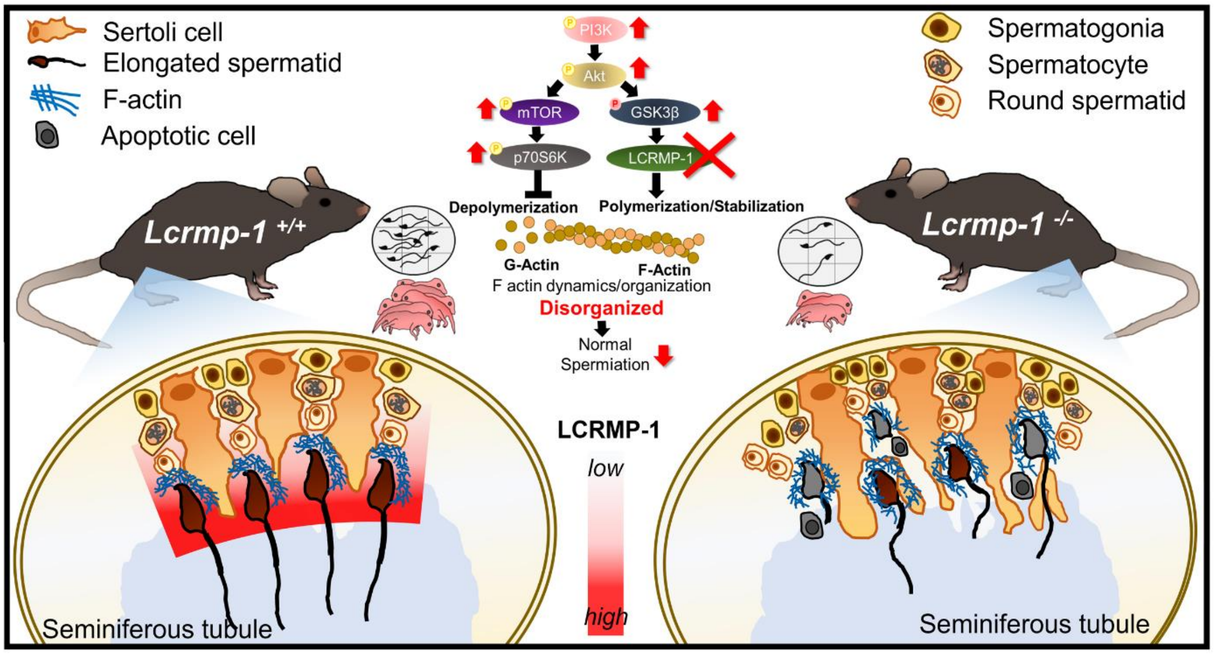

Supplementary Fig. 6 Illustration of unbalancing signaling of F-actin dynamics resulting in reduced fertility.

LCRMP-1 is involved in spermiation by stabilizing F-actin. LCRMP-1 deficiency leads to oligospermia, apoptotic spermatids, reduced fertility with compensatory upregulation (unbalancing) of Akt pathway and disorganization structures of F-actin from stages VII to X during spermatogenesis.

Fig. 1a

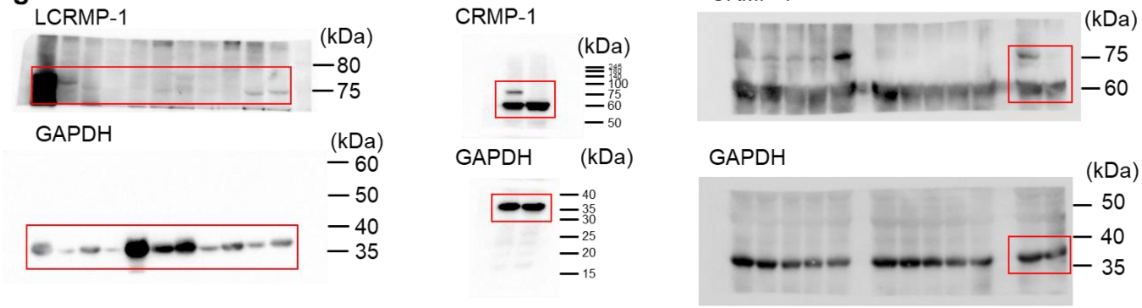

Fig. 1d

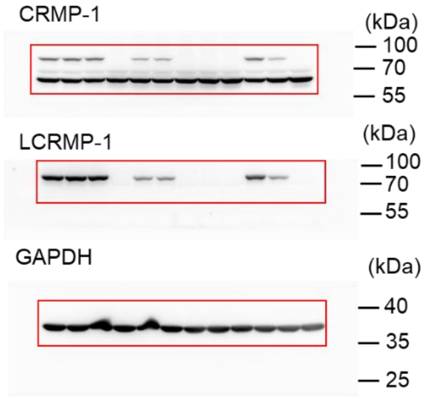

Fig. 3a

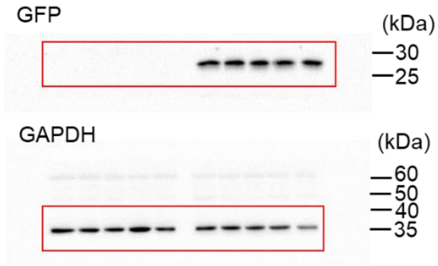

Fig. 4b

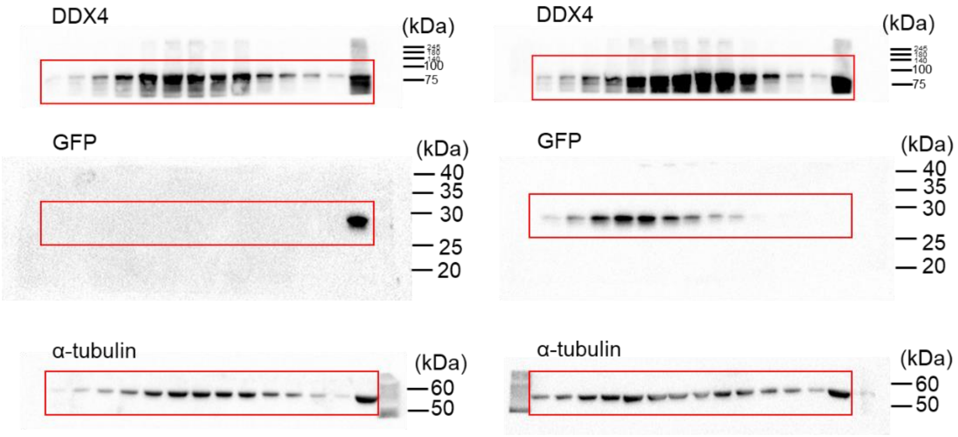

Fig. 7a

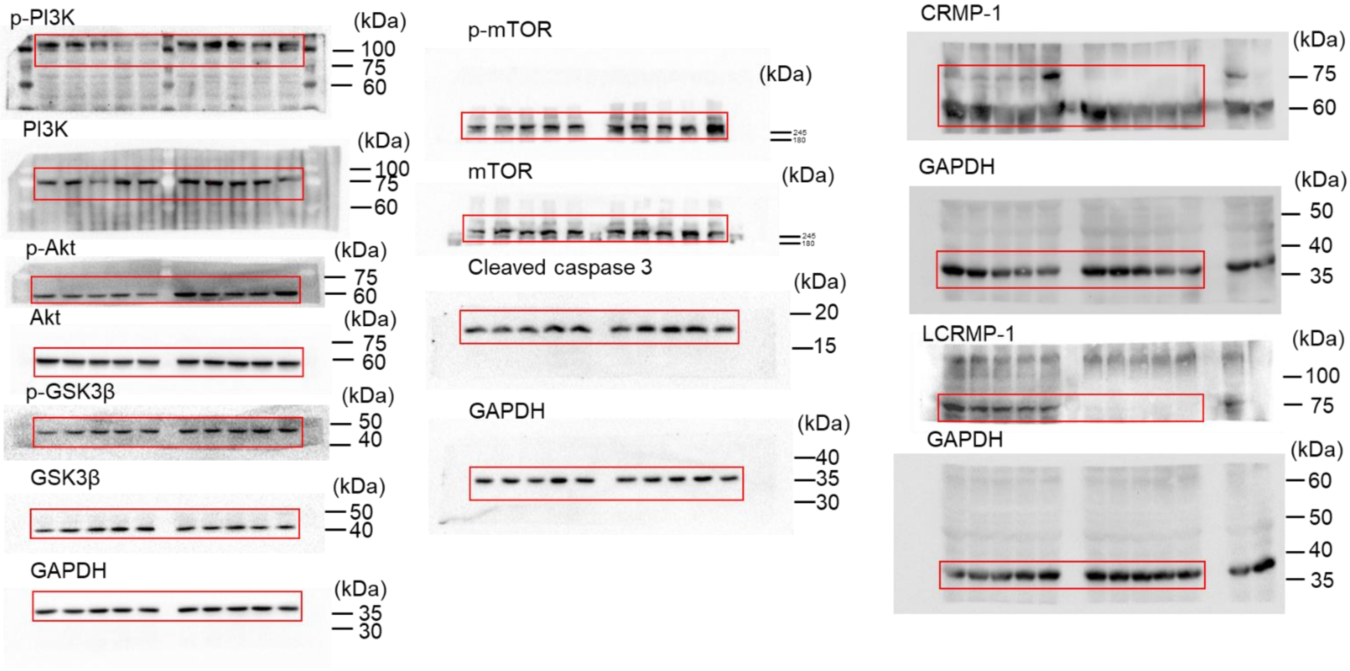
p-PI3K
(kDa)
100
75
60
PI3K
(kDa)
100
75
60
p-Akt
(kDa)
75
60
Akt
(kDa)
75
60
p-GSK3β
(kDa)
50
40
GSK3β
(kDa)
50
40
GAPDH
(kDa)
35
30
p-mTOR
(kDa)
mTOR
(kDa)
Cleaved caspase 3
(kDa)
20
15
GAPDH
(kDa)
40
35
30
CRMP-1
(kDa)
75
60
GAPDH
(kDa)
50
40
35
LCRMP-1
(kDa)
100
75
GAPDH
(kDa)
60
50
40
35

Fig. 7b

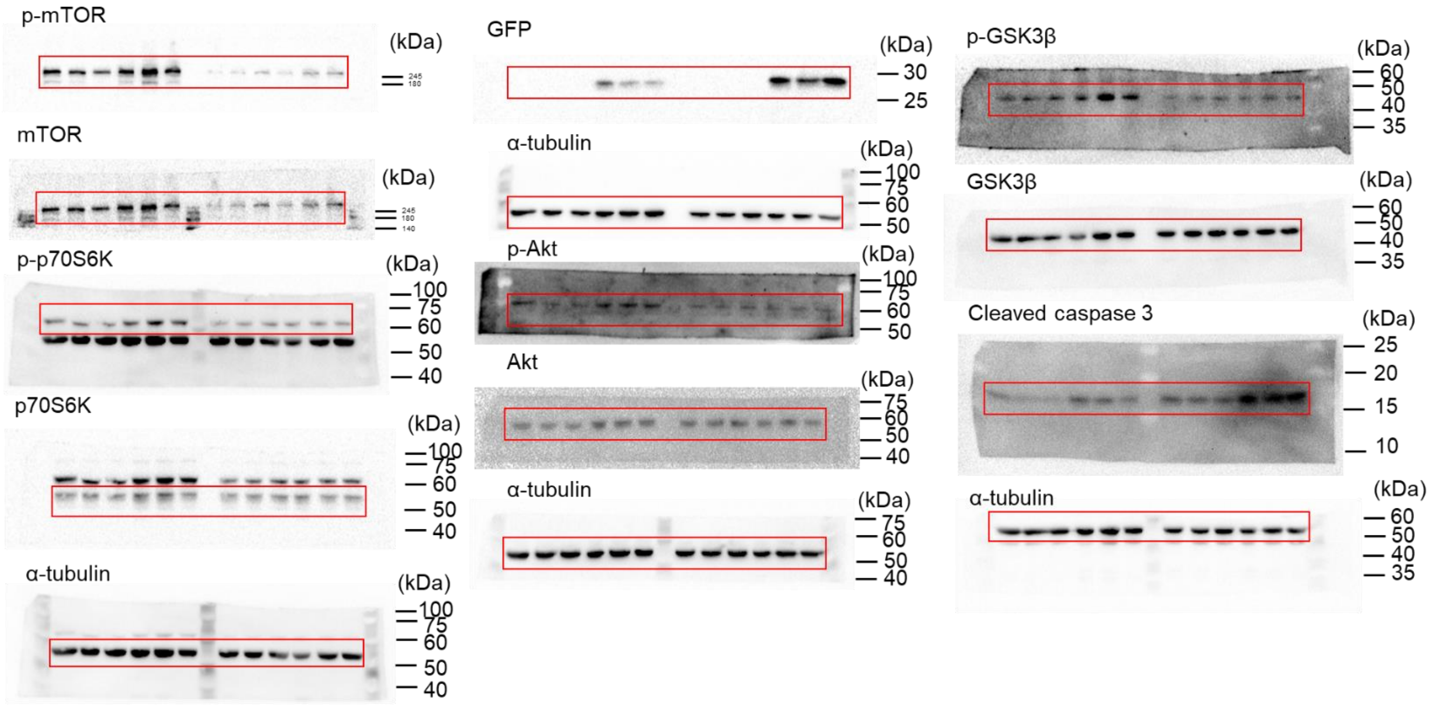
p-mTOR
(kDa)
mTOR
(kDa)
p-p70S6K
(kDa)
100
75
60
50
40
p70S6K
(kDa)
100
75
60
50
40
α-tubulin
(kDa)
100
75
60
50
40
GFP
(kDa)
30
25
α-tubulin
(kDa)
100
75
60
50
p-Akt
(kDa)
100
75
60
50
Akt
(kDa)
75
60
50
40
α-tubulin
(kDa)
75
60
50
40
p-GSK3β
(kDa)
60
50
40
35
GSK3β
(kDa)
60
50
40
35
Cleaved caspase 3
(kDa)
25
20
15
10
α-tubulin
(kDa)
60
50
40
35

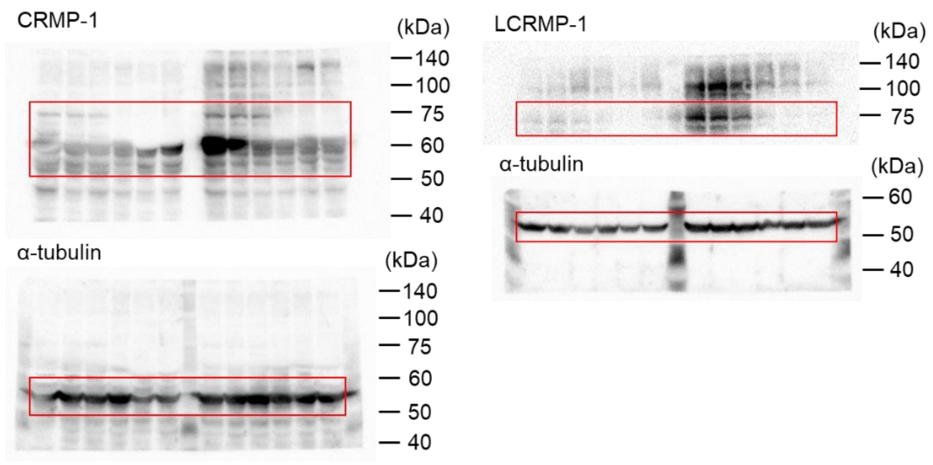

Supplementary Fig. 7 Original Western Blots

The original blots for Figures 1a, d; 3a; 4b; 7a, b.

Supplementary Table 1. Histopathological analysis report of major organs in *Lcrmp-1*$^{+/+}$ and *Lcrmp-1*$^{-/-}$ mice.

Major Organ/Tissue	Histopathological findings of nonneoplastic lesions	Grading schemes of pathological finding[a]					
		Lcrmp-1$^{-/-}$ (N=3)			*Lcrmp-1*$^{+/+}$ (N=3)		
		No. 1	No. 2	No .3	No. 1	No. 2	No. 3
Brain		0	0	0	0	0	0
Heart		0	0	0	0	0	0
Liver	Extramedullary hematopoiesis, focal[b,c]	1	1	1	1	1	1
Kidney	Regeneration, renal tubule, cortex, focal[d]	0	0	0	1	0	0
	Infiltration, mononuclear cell, interstitium, focal	0	1	0	0	0	0
	Mineralization, collecting duct, papilla, focal[e]	0	1	1	1	0	0
Spleen		0	0	0	0	0	0
Lung		0	0	0	0	0	0
Testis	Degeneration, germ cell, seminiferous tubule, focal to diffuse[f]	1	5	5	1	1	1

	Vacuolation, seminiferous tubule, focal[g]	1	1	1	1	1	1
	Atypical residual bodies, seminiferous tubule, focal[h]	1	0	0	1	1	1
	Multinucleated giant cell, seminiferous tubule, focal[i]	0	1	1	0	0	0
Sciatic nerve		0	0	0	0	0	0
Pituitary gland		0	0	0	0	0	0
Spinal cord		0	0	0	0	0	0

[a] Severity of lesions was graded according to the previous study [1]. It was relative to the percentage of the tissue/organ area affected. The scores, "0" means no presence of significant histology change. The degree of lesions stained with H&E was graded from one to five depending on severity: 1= minimal (<1%); 2: slight (1-25%); 3= moderate (26-50%); 4= moderately severe/high (51-75%); 5= severe/high (76-100%). [b] Focal lesion. [c]The hematopoietic cells randomly appeared in the hepatic sinusoids. [d]The necrosis of tubular epithelium followed by regeneration with intact basement membrane. [e]The lesion with dense basophilic granular deposits as a calcification due to systemic calcium and phosphorus imbalance. [f]The lesion with decreased elongating spermatids. Germ cell death can be attributed to disturbances in Sertoli cell regulating the survival of germ cell. [g]The vacuoles within the seminiferous epithelium may result from degenerative changes and the loss of embedded germ cells. [h]The lesions showed abnormally large or clumped residual bodies, which appeared at the luminal surface or resorbed into Sertoli cell cytoplasm. [i]The multinucleated giant cell within the seminiferous epithelium may result from the degeneration of spermatids and spermatocytes.

Supplementary Table 2 Primers utilized for PCR and qPCR.

Primers for genotyping (5'- 3')	
ex1aF	ATG ACA GAC CGC CAG CGT TG
In1aR	CCT GGC CTA TCC TAC AAC CTC
egfpF	ATC ACT CTC GGC ATG GAC GA
Primers for qPCR (5'- 3')	
LCRMP-1 (F)	GGA CAA TGG CCA GAG TGA C
LCRMP-1 (R)	ATT CGC CTC GAT GGT CTT C
CRMP-1 (F)	CAT CAG GGG AAG AAG AGC AT
CRMP-1 (R)	CCA CCA GGA ACA ATC AGG TT
TBP (F)	CCG TGA ATC TTG GCT GTA AAC TTG
TBP (R)	GTT GTC CGT GGC TCT CTT ATT CTC

Supplementary Reference

1 Shackelford, C., Long, G., Wolf, J., Okerberg, C. & Herbert, R. Qualitative and quantitative analysis of nonneoplastic lesions in toxicology studies. *Toxicol Pathol* **30**, 93-96, doi:10.1080/01926230252824761 (2002).
