# Supplementary material for: LCRMP-1 is required for spermatogenesis and stabilises spermatid F-actin organization via the PI3K-Akt pathway
Source: Commun Biol. 2023 Apr 10;6:389. doi: 10.1038/s42003-023-04778-2 (PMC10086033; doi:10.1038/s42003-023-04778-2)
Supplement: Supplementary file 3 — Description of Additional Supplementary Files [file 42003_2023_4778_MOESM3_ESM.pdf]

Description of Additional Supplementary Files

File name: Supplementary Data 1

Description: The raw data of the graphs in the paper.

File name: Supplementary Movie 1

Description: : The supplementary movie for Fig. 2d.
