# Supplementary material for: LCRMP-1 is required for spermatogenesis and stabilises spermatid F-actin organization via the PI3K-Akt pathway
Source: Commun Biol. 2023 Apr 10;6:389. doi: 10.1038/s42003-023-04778-2 (PMC10086033; doi:10.1038/s42003-023-04778-2)
Supplement: Supplementary file 6 — Reporting Summary [file 42003_2023_4778_MOESM6_ESM.pdf]

nature portfolio

Corresponding author(s): Kang-Yi Su

Last updated by author(s): Feb 15, 2023

# Reporting Summary

Nature Portfolio wishes to improve the reproducibility of the work that we publish. This form provides structure for consistency and transparency in reporting. For further information on Nature Portfolio policies, see our Editorial Policies and the Editorial Policy Checklist.

## Statistics

For all statistical analyses, confirm that the following items are present in the figure legend, table legend, main text, or Methods section.

| n/a | Confirmed | |
|---|---|---|
| ☐ | ☒ | The exact sample size (*n*) for each experimental group/condition, given as a discrete number and unit of measurement |
| ☐ | ☒ | A statement on whether measurements were taken from distinct samples or whether the same sample was measured repeatedly |
| ☐ | ☒ | The statistical test(s) used AND whether they are one- or two-sided<br>*Only common tests should be described solely by name; describe more complex techniques in the Methods section.* |
| ☒ | ☐ | A description of all covariates tested |
| ☒ | ☐ | A description of any assumptions or corrections, such as tests of normality and adjustment for multiple comparisons |
| ☐ | ☒ | A full description of the statistical parameters including central tendency (e.g. means) or other basic estimates (e.g. regression coefficient) AND variation (e.g. standard deviation) or associated estimates of uncertainty (e.g. confidence intervals) |
| ☐ | ☒ | For null hypothesis testing, the test statistic (e.g. *F*, *t*, *r*) with confidence intervals, effect sizes, degrees of freedom and *P* value noted<br>*Give P values as exact values whenever suitable.* |
| ☒ | ☐ | For Bayesian analysis, information on the choice of priors and Markov chain Monte Carlo settings |
| ☒ | ☐ | For hierarchical and complex designs, identification of the appropriate level for tests and full reporting of outcomes |
| ☒ | ☐ | Estimates of effect sizes (e.g. Cohen's *d*, Pearson's *r*), indicating how they were calculated |

*Our web collection on statistics for biologists contains articles on many of the points above.*

## Software and code

Policy information about availability of computer code

| | |
|---|---|
| Data collection | Zen(black edition) was used for image acquisition from Zeiss LSM 880 and Zeiss LSM 780 confocal microscope. Olympus Cell Sens Dimension Software was used for Olympus IX83 system. DP Manager(Olympus) Software was used for image capture from Olympus microscope. QuantStudio Design & Analysis Software was used for qPCR acquisition. The chemiluminescent signal of Western blotting was acquired by Image Lab 6.0 Software. |
| Data analysis | OpenCASA software (version 2.0, using imageJ) was used for sperm motility analysis. ImageJ bundled with Java 8 was used to quantify the results of immunofluorescence staining and Western blotting. |

For manuscripts utilizing custom algorithms or software that are central to the research but not yet described in published literature, software must be made available to editors and reviewers. We strongly encourage code deposition in a community repository (e.g. GitHub). See the Nature Portfolio guidelines for submitting code & software for further information.

## Data

Policy information about availability of data

All manuscripts must include a data availability statement. This statement should provide the following information, where applicable:

- Accession codes, unique identifiers, or web links for publicly available datasets
- A description of any restrictions on data availability
- For clinical datasets or third party data, please ensure that the statement adheres to our policy

All data in this study are available within the article and from the corresponding author on reasonable request.

## Human research participants

Policy information about studies involving human research participants and Sex and Gender in Research.

| | |
|---|---|
| Reporting on sex and gender | N/A |
| Population characteristics | N/A |
| Recruitment | N/A |
| Ethics oversight | N/A |

Note that full information on the approval of the study protocol must also be provided in the manuscript.

# Field-specific reporting

Please select the one below that is the best fit for your research. If you are not sure, read the appropriate sections before making your selection.

☒ Life sciences ☐ Behavioural & social sciences ☐ Ecological, evolutionary & environmental sciences

For a reference copy of the document with all sections, see nature.com/documents/nr-reporting-summary-flat.pdf

# Life sciences study design

All studies must disclose on these points even when the disclosure is negative.

| | |
|---|---|
| Sample size | These sample sizes were not predetermined by statistical methods. The sample sizes were determined based on the amount of biological materials. Based on previous publications (PMID: 36131086, 34059773, 32674113) and our pilot tests, the sample sizes in this study are sufficient. |
| Data exclusions | No data were excluded in this study. |
| Replication | The data from mouse experiments was generated in independent biological replicates. |
| Randomization | We performed each experiment with wild-type and Lcrmp-1- deficient mice during the same time for comparison in a same conditions because each group of mice was classified according to genotype. |
| Blinding | There was no blinding in this study due to smaller number of samples each time we perform the experiments. |

# Reporting for specific materials, systems and methods

We require information from authors about some types of materials, experimental systems and methods used in many studies. Here, indicate whether each material, system or method listed is relevant to your study. If you are not sure if a list item applies to your research, read the appropriate section before selecting a response.

## Materials & experimental systems

| n/a | Involved in the study |
| --- | --- |
| ☐ | ☒ Antibodies |
| ☒ | ☐ Eukaryotic cell lines |
| ☒ | ☐ Palaeontology and archaeology |
| ☐ | ☒ Animals and other organisms |
| ☒ | ☐ Clinical data |
| ☒ | ☐ Dual use research of concern |

## Methods

| n/a | Involved in the study |
| --- | --- |
| ☒ | ☐ ChIP-seq |
| ☒ | ☐ Flow cytometry |
| ☒ | ☐ MRI-based neuroimaging |

# Antibodies

Antibodies used

CRMP-1, Cat #sc-365348, Santa Cruz biotechnology, 1:1000 (WB), Cat #PA5-34768, Thermo Fisher Scientific, 1:1500 (WB)
LCRMP-1, Pan SH Lab, 1:10000 (WB)(homemade)
GSK3β, Cat#9315, Cell signaling technology, 1:1000 (WB)
phosphorylated GSK3β, Cat#9336, Cell signaling technology, 1:1000 (WB)
GFP, Cat#2956, Cell signaling technology, 1:1000 (WB, IHC)
cleaved caspase 3, Cat#9661, Cell signaling technology, 1:1000 (WB)
mTOR, Cat#2972, Cell signaling technology, 1:1000 (WB)
phosphorylated mTOR, Cat#2971, Cell signaling technology, 1:1000 (WB)
Akt, Cat#9272, Cell signaling technology, 1:1000 (WB)
phosphorylated Akt, Cat#9271, Cell signaling technology, 1:1000 (WB)
PI3K, Cat#4292, Cell signaling technology, 1:1000 (WB)
phosphorylated PI3K, Cat#4228, Cell signaling technology, 1:1000 (WB)
p70S6K, Cat#2708, Cell signaling technology, 1:1000 (WB)
phosphorylated p70S6K, Cat#9234, Cell signaling technology, 1:1000 (WB)
α-tubulin, Cat#11224-1-AP, proteintech, 1:20000 (WB)
GAPDH, Cat#60004-1-Ig, proteintech, 1:20000 (WB)
DDX4, Cat#ab13840, Abcam, 1:2000, 1:100 (WB, IF), Cat#8761, Cell signaling technology, 1:100(IF)
Goat Anti-Mouse IgG Antibody, peroxidase conjugated, H+L, Cat#AP124P, Sigma-Aldrich, 1:5000 (WB)
Goat Anti-Rabbit IgG H&L (HRP) preadsorbed, Cat#ab7090, Abcam, 1:5000 (WB)
SCP3, Cat#ab97672, Abcam, 1:100 (IF)
SOX9, Cat#H00006662-M01, Abnova, 1:100(IF)
Goat anti-Mouse IgG (H+L) Cross-Adsorbed Secondary Antibody, Alexa Fluor™ 488, Cat# A-11001, Thermo Fisher, 1:500 (IF)
Goat anti-Rabbit IgG (H+L) Cross-Adsorbed Secondary Antibody, Alexa Fluor™ 488, Cat# A-11008, Thermo Fisher, 1:500 (IF)
N-Histofine Simple Stain Mouse MAX PO (M) or (R) secondary antibody, Nichirei Biosciences Inc. Cat#414131F, Cat#l 414141F (IHC)

Validation

CRMP-1, Cat #sc-365348, Santa Cruz biotechnology
RRID:AB_10846086, website: https://scicrunch.org/resolver/AB_10846086
CRMP-1, Cat #PA5-34768, Thermo Fisher Scientific
RRID:AB_2552120, website: https://antibodyregistry.org/search.php?q=AB_2552120
LCRMP-1, Pan SH Lab(Homemade)
Pan SH et al. 2011, PMID: 21747164
GSK3β, Cat#9315, Cell signaling technology
RRID:AB_490890, website: https://antibodyregistry.org/search.php?q=AB_490890
phosphorylated GSK3β, Cat#9336, Cell signaling technology
RRID:AB_331405, website: https://antibodyregistry.org/search.php?q=AB_331405
GFP, Cat#2956, Cell signaling technology
RRID:AB_1196615, website: https://antibodyregistry.org/search.php?q=AB_1196615
cleaved caspase 3, Cat#9661, Cell signaling technology
RRID:AB_2341188, website: https://antibodyregistry.org/search.php?q=AB_2341188
mTOR, Cat#2972, Cell signaling technology
RRID:AB_330978, website: https://antibodyregistry.org/search.php?q=AB_330978
phosphorylated mTOR, Cat#2971, Cell signaling technology
RRID:AB_330970, website: https://antibodyregistry.org/search.php?q=AB_330970
Akt, Cat#9272, Cell signaling technology
RRID:AB_329827, website: https://antibodyregistry.org/search.php?q=AB_329827
phosphorylated Akt, Cat#9271, Cell signaling technology
RRID:AB_329825, website: https://antibodyregistry.org/search.php?q=AB_329825
PI3K, Cat#4292, Cell signaling technology
RRID:AB_329869, website: https://antibodyregistry.org/search.php?q=AB_329869
phosphorylated PI3K, Cat#4228, Cell signaling technology
RRID:AB_659940, website: https://antibodyregistry.org/search.php?q=AB_659940
p70S6K, Cat#2708, Cell signaling technology
RRID:AB_390722, website: https://antibodyregistry.org/search.php?q=AB_390722
phosphorylated p70S6K, Cat#9234, Cell signaling technology
RRID:AB_2269803, website: https://antibodyregistry.org/search.php?q=AB_2269803
α-tubulin, Cat#11224-1-AP, proteintech
RRID:AB_2210206, website: https://antibodyregistry.org/search.php?q=AB_2210206
GAPDH, Cat#60004-1-Ig, proteintech
RRID:AB_2107436, website: https://antibodyregistry.org/search.php?q=AB_2107436

DDX4, Cat#ab13840, Abcam
RRID:AB_443012, website: https://antibodyregistry.org/search.php?q=AB_443012
DDX4, Cat#8761, Cell signaling technology
RRID:AB_2797658, website: https://antibodyregistry.org/search.php?q=AB_2797658
SCP3, Cat#ab97672, Abcam
RRID:AB_10678841, website: https://antibodyregistry.org/search.php?q=AB_10678841
SOX9, Cat#H00006662-M01, Abnova
RRID:AB_535042, website: https://antibodyregistry.org/search.php?q=AB_535042

## Animals and other research organisms

Policy information about studies involving animals; ARRIVE guidelines recommended for reporting animal research, and Sex and Gender in Research

| | |
|---|---|
| Laboratory animals | 6-8 weeks and 6-7 months<br>Lcrmp-1 +/+ and Lcrmp-1 -/-(C57BL/6) |
| Wild animals | This study did not involve wild animals. |
| Reporting on sex | Our findings apply to only male mice. The expression level of LCRMP-1 was significantly higher in the testes (male reproduction) rather than ovaries (female reproduction). |
| Field-collected samples | Our study did not involve field-collected samples. |
| Ethics oversight | All animal experiments and protocols were approved by the Institutional Animal Care and Use Committee (IACUC) of National Taiwan University College of Medicine with IACUC number 20201058. |

Note that full information on the approval of the study protocol must also be provided in the manuscript.
